# Host innate immune systems gather intel on invading microbes via pathogen-derived extracellular vesicles

**DOI:** 10.1016/j.vesic.2024.100043

**Published:** 2024-05-17

**Authors:** Geneva N. Kwaku, Rebecca A. Ward, Jatin M. Vyas, Hannah Brown Harding

**Affiliations:** a Division of Infectious Diseases, Department of Medicine, Massachusetts General Hospital, Boston, MA, USA; b Department of Medicine, Harvard Medical School, Boston, MA, USA; c Broad Institute of MIT and Harvard, Cambridge, MA, USA

**Keywords:** Extracellular vesicles, Innate immunity, Inflammatory pathways, Therapeutics

## Abstract

Extracellular vesicles (EVs) are membrane-bound vesicles released into the extracellular milieu from various cell types including host cells and pathogens that infect them. As carriers of nucleic acids, proteins, lipids, metabolites, and virulence factors, EVs act as delivery vehicles for intercellular communication and quorum sensing. Innate immune cells have the capacity to intercept, internalize, and interpret ‘messages’ contained within these EVs. This review categorizes the ability of EVs secreted by bacterial, parasitic, and fungal pathogens to trigger both pro- and anti-inflammatory innate immune responses in the host. Understanding molecular pathways and inflammatory responses activated in innate immune cells upon pathogen-derived EV stimulation is critical to gain insight into potential therapeutics and combat these infectious diseases.

## 1. Introduction

Extracellular vesicles (EVs), or exosomes, were first identified nearly two decades ago as important mediators of intracellular communication that could shuttle RNA between mast cells.^1^ Since then, studies have shown that EVs can be released and subsequently received by a multitude of cell types, including between pathogen and host cells. With respect to host-pathogen relationships, current evidence demonstrates that cells of the innate immune system intercept these vesicles traveling between pathogens prior to initial physical contact with the invading microorganisms, eliciting pro-inflammatory responses by the host to fight infection.^2^ Interestingly, certain pathogenic species secrete EVs that conversely activate anti-inflammatory responses in immune cells, ultimately making the host more susceptible to disease.^3^ This pathogen- and cell-dependent response between host innate immune cells and the EVs secreted by bacterial, parasitic, and fungal pathogens will be reviewed.

Diverse pathogens produce EVs that can be intercepted by host innate immune cells (*e.g*., macrophages, dendritic cells) and epithelial cells. Gram-negative and Gram-positive bacterial pathogens secrete EVs referred to as outer membrane vesicles (OMVs) and membrane vesicles (MV), respectively.^4^,^5^ OMVs bud off from the outer membrane of Gram-negative bacteria, meanwhile, it is believed that Gram-positive bacteria secrete MVs through endolysin-mediated pores in the thick peptidoglycan layer.^4^,^6^ We will refer to both types of EVs generally as bacterial EVs in this review and will discuss the specific and dynamic effects bacterial EVs have on innate immune cells. These findings will be summarized in Table 1.

The lesser studied parasitic pathogens also produce EVs. Since parasites live on or within host organisms as a means of survival, the EVs they produce tend to modulate the host immune system in a variety of ways to encourage pathogen growth or homeostasis. EV-producing parasites can be sub-classified into either protozoa or helminths and will be reviewed as such. Parasitic protozoa are unicellular organisms that constitute a major health burden as they are the causative agents of many diseases worldwide.^7^ This review will describe the inflammatory responses induced in innate immune cells in response to protozoan parasites, with the findings summarized in Table 2. Parasitic worms, or helminths, are disease-causing multicellular parasites that secrete an immense number of EVs in a short time frame. For example, a singular *Brugia malayi* secretes approximately 19,000 EVs in 24 h. An infected individual hosts thousands of microfilariae, the early-stage worm, per milliliter of blood, providing just one example of the incentive for studying the immunomodulatory effects of parasite-derived EVs.^8^ The effects that helminth EVs have on cells of the innate immune system will be summarized in Table 3.

Like bacteria and parasites, pathogenic fungi secrete EVs used for cell-cell communication. Interestingly, fungal EVs have been studied largely in the context of fungal biofilms, as these EVs potently impact biofilm growth and formation.^9^,^10^ The formation of microbial biofilms in the host can be detrimental, as they are often resistant and impermeable to drugs and, therefore, difficult to treat. Biofilm regulation by EVs provides further motivation for understanding pathogenic, specifically fungal-derived, EVs and their interactions with the host immune system.^11^ Evidence of fungal-derived EVs on host innate immune systems will be summarized in Table 4.

The size of pathogen-derived EVs ranges widely both between unique species and within them, with some of the smallest EVs recorded as 10 nm in diameter from the bacterium *Xanthomonas campestris* and the largest as 600 nm from protozoan parasite *Entamoeba histolytica*.^12^,^13^ Most pathogen-derived EVs, however, fall within the 100–250 nm diameter range. EVs carry a variety of different biomolecules, including lipids, nucleic acids, proteins, and virulence factors.^14^ The cargo is intended for intraspecies intercellular communication; however, during infection, these effector proteins and signaling molecules become intercepted and decoded by host immune cells that encounter pathogenic EVs prior to physical interaction with the etiologic agent. Specific cargo like miRNA sequences or virulent proteins remain essential for this immunomodulation of the host upon EV stimulation, the details of which will be covered in this review.

The ability of immune cells to sense and respond to EVs secreted by pathogens offers key advantages in the host’s capacity to fight infection. Such proficiency suggests the following scenario: a pathogen – a fungal organism, for example – invades a host and releases EVs to communicate with other fungi about the local environment. Macrophages and other innate immune cells intercept these inter-fungal messages, decipher them, and subsequently elicit a robust pro-inflammatory immune response to combat the invading microorganism. Interception of pathogen-derived EVs primes the innate immune system to initiate antimicrobial pathways even before physical contact of the microorganism itself with the immune cells. Gathering intel on the fast-approaching infectious agent through EV capture provides additional time to initiate defenses against it, a scheme likely advantageous to the health of the host. Ultimately, understanding how the innate immune system reacts to pathogen-derived EVs offers insight into their potential as therapeutics or vaccines to alleviate the global burden of infectious diseases.

## 2. Bacteria-derived EVs have pro-inflammatory effects on host innate immunity

### 2.1. Pro-inflammatory cytokine release

The host immune system teeters between pathogenic hyperinflammation and weak, ineffectual immune responses while striving to maintain homeostasis. A myriad of internal and external factors such as stress, autoimmunity, and exposure to pathogens may disrupt this balance. Many bacterial pathogens encountered by the host shift the immune response to induce pro-inflammatory cytokines and signaling pathways to clear the infection. In the last decade, researchers have identified that EVs derived from a variety of bacterial species induce pro-inflammatory cytokine and chemokine release from murine macrophages *in vitro*. For example, EVs secreted by the Gram-positive bacteria *Lactiplantibacillus plantarum* trigger IL-1β and IL-6 release, while *Staphylococcus aureus* EVs induce IFNβ, IL-6, TNFα, CCL2, with many of these responses occurring in a toll-like receptor (TLR)-dependent manner.^15^,^16^,^17^ This immune response is not specific to EVs from those bacteria that lack an outer membrane, as the Gram-negative bacterium *Francisella tularensis* produces EVs that induce pro-inflammatory cytokines in bone marrow-derived macrophages (BMDMs) in a time- and dose-dependent manner.^2^ Interestingly, infection of BMDMs by the whole bacterium *F. tularensis* does not stimulate these pro-inflammatory cytokines, suggesting that EVs are essential for the stimulation of an innate immune response in the host upon pathogenic infection.^2^

EV-induced pro-inflammatory responses extend beyond traditional innate immune cells. Epithelial cells, the first cell barrier encountered by many bacterial pathogens and secreted EVs invading the host, also internalize and respond to bacterial EVs. Intestinal epithelial cells (IECs) have been studied in the context of the immune response to pathogenic EVs due to their constant exposure to microorganisms, including foodborne pathogens and commensal bacteria.^18^ EVs from the Gram-positive bacteria *Listeria monocytogenes*, often found in contaminated food, trigger an upregulation of genes involved in the TNF and IL-17 chemokine signaling pathways in Caco-2 IECs.^19^ Similarly, *Fusobacterium nucleatum*, a bacterium associated with periodontal and colorectal diseases, secretes EVs that induce the production of IL-8 in T84 IECs.^20^

Bacterial EVs also provoke pro-inflammatory cytokines *in vivo*. Indeed, cytokines RANTES, MCP-1/JE, and CXCL10 are produced in the blood of mice after intravenous injections of EVs secreted by *Streptococcus pneumonia*, the respiratory tract pathogen responsible for most of the pneumonia global burden. Furthermore, these mice displayed clinical signs of arthritis in knee joints after local injection of *S. aureus* EVs in a TLR2-dependent manner.^17^,^21^ This arthritis, characterized by swelling of the joints, is caused by inflammation, revealing the robust pro-inflammatory properties of these EVs.

### 2.2. Increased innate immune cell activation

Bacterial EVs have proven substantial *in vitro* and *in vivo* effects on innate immune cell recruitment and activation. Recruitment of macrophages and natural killer cells *in vivo* often occurs in a dose-dependent manner in mice locally inoculated with bacterial-derived EVs.^21^ The phagocytic capacity of innate immune cells can be altered by bacterial EVs as well. For example, the priming of both human primary macrophages and immortalized mouse macrophages with *S. pneumoniae* EVs increases the phagocytic capacity of the whole bacterium during infection. Interestingly, the bacteria phagocytosed by macrophages in an *S. pneumoniae* EV-primed manner demonstrate greater survival than other phagocytosed bacteria.^21^ These data indicate that EVs from some bacterial species condition macrophages to improve bacterial survival during infection.

### 2.3. Upregulation of pro-inflammatory signaling

Pro-inflammatory responses induced by bacterial-derived EVs in innate immune cells frequently result from NFκB signaling activation via TLRs such as TLR2. In this way, innate immune cells are capable of internalizing bacteria-derived EVs and activating signaling to promote inflammation and pathogen clearance. NFκB signaling is activated in both immortalized murine and primary human macrophages following stimulation by *S. pneumoniae* EVs.^21^ NFκB activation occurs in epithelial cells as well, specifically in IECs upon *L. monocytogenes* EV and *F. nucleatum* EV exposure, the latter occurring in a TLR2-and dyna-min-dependent manner.^19^,^20^ Other cell types, such as HEK293 cells, activate NFκB signaling upon bacterial-derived EV exposure (*e.g*., *L. plantarum* EVs).^15^ Furthermore, EVs from *Gardnerella vaginalis* and *Mobiluncus mulieris* activate NFκB signaling in human epithelial cells, while *Escherichia coli* EVs do so in human macrophages.^22^,^23^ The EVs from *E. coli* additionally activate the pro-inflammatory NLRP3 pathway in human macrophages and the stress-activated SAPK/JNK signaling cascade in murine neutrophils.^23^ Another pro-inflammatory signaling pathway, the cGAS-STING pathway, is activated in response to bacteria-derived EVs. EVs from the Gram-negative bacterium *Pseudomonas aeruginosa* activate this pathway in adipose tissue-derived mesenchymal stem cells (ASCs) as evidenced by upregulation of essential pathway components cGAS, p-TBK1, and p-IRF3, as well as pathway output IL-7, upon EV stimulation.^24^ The evidence that different bacterial EVs stimulate many of the same essential pro-inflammatory signaling cascades in various immune cells reveals a common potential target for immunotherapies.

## 3. Bacteria-derived EVs have anti-inflammatory effects on host innate immunity

### 3.1. Coordinated anti-inflammatory cytokine release

Host epithelial cells often respond to EVs derived from some bacterial species with an anti-inflammatory immune response through the induction of anti-inflammatory cytokines. Some bacterial species signal to host cells to subvert an immune response, suppressing proinflammatory cytokines, and allowing for a successful and potentially fatal bacterial infection. For example, IECs increase expression of antiinflammatory pathway activator miR-146a after stimulation with EVs isolated from *Vibrio cholerae*, the bacteria commonly found in contaminated food and water. This induction is independent of the presence of the prominent virulence factor: *V. cholerae* cytolysin (VCC). Interestingly, delivery of VCC in EVs did not induce upregulation of proinflammatory cytokines IL-1β and CCL20 in IECs whereas soluble VCC alone did.^3^ This differential response suggests that packaging toxic cargo in EVs is sufficient for dampening their inflammatory effects— a method utilized by bacteria to avoid generating a primary immune reaction at the epithelium, allowing increased pathogen survival and enhanced disease states.

Rarely do pathogen-derived EVs initiate an anti-inflammatory response alone, but more commonly induce anti-inflammatory cytokines and effector functions in conjunction with inflammation. For example, EVs derived from *L. plantarum*, *P. aeruginosa*, and *F. tularensis* all cause the release of the anti-inflammatory cytokine, IL-10, in coordination with other pro-inflammatory cytokines including IL-1β, IL-6, IL-8, and CXCL1, indicating a mixed response from host cells upon bacterial EV exposure and revealing the complexity of EV-driven innate immunity.^2^,^15^,^25^

### 3.2. Altered immune cell activation type and antigen-presenting capabilities

The complexity in responses elicited by bacteria-derived EVs is enhanced when considering the ability of EVs to simultaneously suppress many pro-inflammatory responses and cell types. Many bacterial pathogens secrete EVs that affect immune cell activation (*i.e*., antigen-presenting capabilities) resulting in dampening of the immune system. For example, *S. pneumoniae* EVs injected intravenously into mice induce a 5-fold total increase of M2-activated macrophages in the blood and spleen.^21^ Importantly, 2-activated macrophages are associated with anti-inflammatory responses contrary to their M1-activated counterparts which are mostly coupled with a pro-inflammatory response. EVs derived from *Helicobacter pylori* also led to an increase in CD206, the marker for M2 macrophage polarization, on murine macrophages and a decrease in CD86, a marker for M1 polarization, providing further evidence for impacts on immune cell differentiation by bacteria-derived EVs.^26^ In addition to modulating macrophage activation type, bacterial EVs may suppress innate immune cell killing capacity by downregulation of Major Histocompatibility Complexes (MHCs), the site of antigen presentation on immune cells. *P. aeruginosa* EVs suppress the gene expression and protein levels of many MHC-associated molecules on lung macrophages, which are among the first lines of innate immune defense against pathogens in the human airway.^25^ Such immunosuppression results in a weakened capacity for pathogen clearance and describes a bacterial strategy for immune evasion via EVs.

### 3.3. EV-induced changes in host cell survival

Bacterial EVs also function as potent modulators of host cell survival via specialized cargo delivered to specific structures or processes in the host cell that results in host cell death. For example, *Neisseria gonorrhoeae* secretes EVs that deliver the outer membrane protein PorB to BMDMs as protein cargo. PorB then localizes to and damages the mitochondrial network of the host cell inducing high levels of cytochrome C and subsequently activates host cell death. Along with this potent mitochondrial damage, these EVs rupture BMDM plasma membranes leading to cell death.^27^ Importantly for the bacteria, both of these mechanisms of EV-induced cell death lead to increased pathogen survival. Evidently, the diverse cargo carried within bacterial EVs alters host immune cell survival to ensure that pathogenic bacteria can escape and persist within the host immune system.

## 4. Protozoan parasite-derived EVs stimulate the host immune system

### 4.1. Pro-inflammatory cytokine and free radical release

Innate immune cells internalize EVs from protozoan parasites and induce pro-inflammatory signaling cascades to combat infection. Macrophages upregulate pro-inflammatory cytokines when stimulated with EVs isolated from *Trypanosoma cruzi* (TNFα, IL-6), *Giardia duodenalis* (TNFα, IL-6, IL-1β), and *Toxoplasma gondii* (mRNA expression TNFα and iNOS).^29^,^30^,^31^ Macrophages also demonstrate increased nitric oxide (NO) and urea production, two products of inflammatory signaling, in response to *Trypanosoma brucei* EVs.^32^ IECs exhibit an increase in some of the same pro-inflammatory cytokines, like IL-1β and IL-6, induced in macrophages when stimulated with EVs from the protozoan parasite *Eimeria falciformis*, along with others including IL-17 and IL-18.^33^ Additionally, stimulation of human neutrophils with *E. histolytica* EVs results in a significant increase in reactive oxygen species (ROS).^13^ This specific pattern of cytokine induction and free radical production is consistent with a pro-inflammatory phenotype and evidence for the ability of parasite-derived EVs to activate the host’s innate immune response.

### 4.2. Altered MHC expression and presentation

Another way protozoan parasite EVs modulate the host immune response occurs through altering antigen-presenting capacities of immune cells. The antigen-presenting complexes of mouse macrophage cell line P388D1 were analyzed as a result of exposure to *T. brucei* EVs. Class I and class II MHCs cell surface expression increased after EV exposure, which is interestingly the opposite phenotype upon whole parasite exposure.^32^ Thus, macrophage interception and interpretation of the signals carried within parasite-derived EVs is an essential priming step for the inevitable infection, as these innate immune cells might respond more vigorously to EVs than to the parasitic organisms themselves.

### 4.3. Activation of pro-inflammatory signaling

The MAPK signaling pathway is induced by some protozoan-derived EVs in host innate immune cells. EVs from *Naegleria fowleri* trigger MAPK signaling in microglial cells, as do *T. cruzi* EVs in mouse macrophages, demonstrated by activation of several essential pathway components including proteins ERK1/2, p38, and JNK.^34^,^29^ *G. duodenalis* EVs similarly activate pro-inflammatory cytokine transcription and secretion via MAPK signaling in murine macrophages evidenced by phosphorylation of p38 and ERK after EV treatment. Molecular inhibition of these proteins significantly downregulates IL-1β, IL-6, and TNFα production following EV stimulation, demonstrating activation of the MAPK pathway in response to these EVs.^35^ EVs from *G. duodenalis* additionally lead to NLRP3 inflammasome activation in murine macrophages. Like many of the aforementioned bacterial EVs, these parasite-derived EVs also activate the NFκB signaling pathway responsible for pro-inflammatory cytokine production. The MAPK and NFκB signaling pathways are orchestrated signaling cascades highly conserved across many cell types. As such, it is unsurprising that they are activated by a variety of pathogenic EVs in many cell types, but more research is needed to explore specific factors that lead to the activation of one over the other. Additionally, the highly conserved JAK-STAT signaling pathway is activated in microglial cells following exposure to *N. fowleri-derived* EVs, as evidenced by phosphorylation of pathway components JAK-1 and STAT3.^34^

## 5. Protozoan parasite-derived EVs dampen the immune system

### 5.1. Modulation of cytokine release

Similarly to bacterial-derived EVs, some parasite species secrete EVs that lessen the immune responses of innate immune cells to improve parasite survival within the host. The EVs released by protozoan parasite *Trypanosoma evansi* downregulate the production of pro-inflammatory cytokines IL-12p40, IL-6, and TNFα in mouse BMDMs via the TLR2-AKT pathway.^36^ In this way, the EVs are secreted by the parasite for its advantage to enhance the chance of survival within the host without immunological detection. Likewise, EVs from *Leishmania infantum* help the parasite survive in the host by upregulating the production of anti-inflammatory cytokine IL-10 while simultaneously downregulating pro-inflammatory cytokines.^37^ The ability of these parasite-derived EVs to disrupt traditional inflammatory responses is not shared by the whole organism, making this EV-specific anti-inflammation phenotype especially intriguing.

### 5.2. Altered innate immune cell function, survival, and signaling

In an attempt to evade immune detection, protozoan-derived parasites also secrete EVs that modulate host cell function and survival. Specifically, protozoan-derived EVs regulate host killing capacity through the inhibition of oxidative bursts and NET release by human neutrophils, a function normally responsible for trapping microbes.^13^ EVs from protozoan parasites can be so potent as to cause not only innate immune cell damage but also death, which forcefully dampens any immune responses to parasitic infection. For example, mRNA expression of NLRP6 and caspase 11, both components of the inflammasome pathway and modulators of programmed host cell death, are upregulated in mouse IECs upon *E. falciformis* EV exposure. Murine IECs treated with *E. falciformis* EVs also exhibit increased lactate dehydrogenase (LDH) production, a marker for proinflammatory mouse IEC death.^33^

The AKT signaling pathway, which is implicated in cell survival, can be activated in murine macrophages by EVs produced by *G. duodenalis*. Interestingly, activation of this pathway by *G. duodenalis* EVs negatively regulates the pro-inflammatory cytokines released via MAPK activation. The observation that *G. duodenalis* EVs activate these conflicting pathways illustrates the balance host cells must maintain between the immune response extremes in response to pathogenic EVs.^35^

## 6. Helminth-derived EVs stimulate the immune system

### 6.1. Pro-inflammatory cytokine and free radical release

Like protozoan parasites, many helminth parasite EVs induce mouse macrophages to release pro-inflammatory cytokines. *Clonorchis sinensis* and *B. malayi* EVs induce release of TNFα/IL-6 and TNFα/IL-6/MCP-1/MIP-2/G-CSF/LIX/RANTES, respectively.^38^,^39^ Human umbilical vein endothelial cells exposed to *Schistosoma mansoni* EVs also upregulate genes coding for pro-inflammatory cytokines. Furthermore, *in vivo* experiments have shown that hepatic macrophages of *C. sinensis*-EV treated mice produced significantly increased TNFα compared to control mice.^38^,^40^

### 6.2. Increased activation of innate immune cells

To neutralize helminth infection, immune cells become classically activated to induce innate immune activation, polarization, and downstream pathogen processing. EVs from *C. sinensis*, the worm that causes liver and bile duct pathophysiology, are internalized by hepatic murine macrophages *in vivo* and subsequently activate M1 macrophages, as detected by CD68^+^CD86^+^ immunofluorescence pro-inflammatory markers.^38^ *B. malayi* EVs also induce M1 macrophage activation, which is historically associated with the induction of pro-inflammatory responses upon antigen internalization.^39^ The helminth EV-induced polarization of these innate immune cells skews the host response towards an inflammatory phenotype that clears the corresponding infection quickly.

## 7. Helminth-derived EVs dampen the immune system

### 7.1. Downregulation of pro-inflammatory cytokines

While the EVs of some parasitic species induce pro-inflammatory cytokines in macrophages, others specifically downregulate the induction of pro-inflammatory cytokines in murine macrophages, such as the EVs from *Heligmosomoides polygyrus*, an intestinal helminth commonly found in rodents.^41^ Other helminth-derived EVs induce an anti-inflammatory immune response *in vivo* as evidenced by downregulation of pro-inflammatory cytokines in cultured colon tissues of *Nippostrongylus brasiliensis* and *Trichinella spiralis* EV-treated mice with induced colitis.^42^,^43^ EVs from these species that suppress the pro-inflammatory responses are used by the parasite to dampen immune responses and allow for the parasitic infection to persist.

### 7.2. Decreased innate immune cell activation and signaling

EVs from some helminth parasites dampen the immune response to parasitic infection by promoting the activation of M2 macrophage polarization and targeting the NFκB pathway causing a dampening of the immune response. Immunohistochemical tissue staining of colons from colitis-induced mice treated with *T. spiralis* EVs revealed the inhibition of M1 activation of macrophages, meanwhile expression of M2 macrophage markers was much higher. Furthermore, pretreatment with *T. spiralis* EVs in colitis-induced mice resulted in reduced phosphorylation of ERK1/2, an integral protein in the MAPK signaling pathway, and decreased NFκB expression than in control groups.^43^

Classic metabolic signaling cascades, like the mTOR pathway, are also impacted in host cells in response to helminth EVs. mTOR signaling involves a multitude of proteins that regulate cell proliferation and immune metabolism. EVs derived from the microfilarial stage of the *B. malayi* parasite contain numerous miRNAs known to target the mTOR signaling pathway, including miR-100, miR-7, and miR-71. In the human monocyte THP-1 cell line, mTOR phosphorylation decreases in the presence of microfilarial stage of *B. malayi* EVs, downregulating the mTOR signaling pathway. The extent to which mTOR phosphorylation becomes decreased is even greater than the known mTOR inhibitor, rapamycin, revealing the potent potential of helminth EVs.^8^ Downregulation of the mTOR pathway influences host catabolism and anabolism, suggesting the immunomodulatory capacity of helminth-derived EVs in impacting host cell metabolism, a function critical for the health of the host.

The evidence that innate immune cells contribute to a dampened immune response upon interception of inter-pathogen EVs reveals the double-edged sword of this phenomenon; while immune cells can gain intel on and subsequently induce an immune response against infectious agents upon internalization of pathogen EVs, there is no escaping the cargo that is carried within them. Thus, intercepting pathogen-derived EVs introduces the risk of welcoming cargo into innate immune cells that may be potent enough to dampen immunity before they can fully fight back.

## 8. Fungal-derived EVs stimulate the host immune system

### 8.1. Host innate immune cells production of cytokines and free radicals

Pathogenic fungal species release EVs primarily to communicate with other members of a fungal community for the growth of biofilms or fungal mats within host organs.^9^ However, the signals carried within these EVs stimulate pro-inflammatory immune responses in a variety of host innate immune cells that lead to enhanced clearance of the fungal pathogen. For example, *Candida albicans* EVs induce pro-inflammatory cytokine release from murine macrophages and dendritic cells, such as IL-12, TGF-β, TNFα, and CCL2, in addition to free radicals like NO,^9^ while IL-8 and TNFα production and IL-10 suppression occurs in human macrophages.^44^ EVs generated by the closely related *Candida auris* similarly stimulate dendritic cells to produce IL-6.^45^ *Aspergillus flavus* and *Aspergillus fumigatus*, the filamentous fungi that cause invasive and deadly aspergillosis in immunocompromised individuals, also secrete EVs that induce pro-inflammatory cytokine release of TNFα, IL-6, and IL-1β, as well as free radicals like NO, in a dose-dependent manner in macrophages.^46^ EVs from *Candida glabrata*, *Candida tropicalis*, *and Candida parapsilosis* stimulate human macrophages to upregulate TNFα and IL-8 production.^47^ The dimorphic fungus *Paracoccidioides brasiliensis* stimulates TNFα, IL-6, MCP-1, and NO in murine macrophages while upregulating circulating IL-6, IFNγ, MCP-1, and IL-6 in an *in vivo* murine model.^48^ EVs from *Talaromyces marneffei* also upregulate ROS and pro-inflammatory cytokines in both murine and human macrophages.^49^ This pro-inflammatory response occurs such that the immune system can better fight the fungal infection; a prior inoculation of *C. albicans*, *Cryptococcus neoformans*, and *A. flavus* in the wax moth model of infection using *Galleria mellonella* larvae resulted in a protective effect against the subsequent, respective fungal infections.^46^,^50^,^51^

### 8.2. Increased activation and phagocytic capacity of innate immune cells

Innate immune cells that have encountered fungal EVs often present increased expression of MHCs and other hallmarks of activation. For example, dendritic cells display upregulation of CD86 and class II MHC molecules after *C. albicans* EV treatment.^52^ Additionally, *C. auris* and *C. albicans* EVs have the capacity to activate bone marrow-derived dendritic cells as stimulation with these EVs results in greater expression of class II MHC, CD80, and CD86.^45^ *A. flavus* and *T. marneffei* both release EVs that stimulate M1 macrophage polarization.^46^,^49^ Immune cell killing capacity and pathogen clearance can also be regulated by internalized fungal EVs. EVs from *C. albicans* can influence macrophages and neutrophils to increase phagocytic and fungicidal activity against subsequent infection by the whole *C. albicans* organism.^53^ In an *in vitro* model using BMDMs, *A. flavus* releases EVs that increase the ability for host cells to clear a subsequent fungal challenge.^46^ Additionally, macrophages and neutrophils display increased killing capacity against the whole pathogenic fungus after pre-treatment with *A. fumigatus* EVs, as do macrophages in response to EVs from *C. neoformans* and *T. marneffei*^54^,^49^,^55^. Thus, innate immune cells are notably skilled at deciphering the EVs released from fungal pathogens and responding through the expansion of functional and phagocytic capacity.

### 8.3. Activation of pro-inflammatory pathways

Interestingly, the interaction between EVs from select fungal pathogens and macrophages can cause the host cells to release their own EVs for pro-inflammatory signaling. For example, macrophages previously exposed to EVs from *C. neoformans* release EVs that trigger p53 and mTOR signaling activation in distant macrophages to fight infection.^56^ Another pro-inflammatory pathway activated by fungal EVs is the cGAS-STING pathway. There is recent evidence that *C. albicans* EVs deliver DNA to macrophages resulting in the activation of the DNA-dependent cGAS-STING signaling pathway, eliciting pro-inflammatory cytokines including IFNβ and the protein viperin, the product of an early interferon-stimulated gene (RSAD2).^57^ The significant increase of IFNβ and viperin induced in wildtype macrophages upon exposure to *C. albicans* and *C. auris* EVs is absent in *cGas*^−/−^ and *Sting*^−/−^ macrophages, indicating the activation of this specific inflammatory pathway. Furthermore, the cGAS-STING pathway is essential for clearance of *C. albicans* infection in mice; wildtype mice infected with a lethal dose of *C. albicans* have significantly decreased survival and increased fungal burden in the kidneys compared to *Sting*^−/−^ mice.^57^ Since EVs stimulate this DNA-dependent pathway, *C. albicans* clearance may be mediated by secreted EVs, demonstrating the importance of host immune cells intercepting these signaling molecules and responding accordingly.

## 9. Fungal EVs dampen the host immune system

Few species of fungi have been shown to induce anti-inflammatory responses in a host upon stimulation by fungal EVs. These antiinflammatory effects manifest mostly in the regulation of cytokine release. For example, EVs from *C. neoformans*, *P. brasiliensis*, *and T. marneffei* induce the anti-inflammatory cytokine IL-10 in macrophages.^48^,^49^,^55^ EVs from *P. brasiliensis* additionally downregulate NO and IL-6 production, two products typical of pro-inflammatory signaling, in the lungs of mice in an *in vivo* experiment.^48^ Whether the lack of evidence supporting fungal EVs dampening host innate immune responses is due to the infancy of the field or the inherent characteristics of these particular EVs, these findings are especially intriguing.

## 10. Clinical relevance of pathogen-EV induced innate immunity

As laid out in this review, the ability of pathogen-derived EVs to trigger innate immune responses can be detrimental or beneficial to the host depending on the severity of response to infection. Thus, innate and adaptive immune triggering mechanisms have been repurposed for novel cancer treatments, vaccines, or novel therapeutics. Furthermore, the ability of EVs to enter systemic circulation, disseminate throughout the body, and be detected in diverse organs and bodily fluids has elicited investigation into EV-based therapeutics and deliverables.

### 10.1. Pathogen-derived EVs as novel cancer therapeutics

EVs from diverse bacterial species have been studied in the context of cancer immunotherapy with much success. EVs isolated from Gram-negative bacteria injected intravenously into mice significantly suppress tumor growth and tumor rebound. Specifically, EVs isolated from an endotoxin-free mutant of *E. coli* (msbB^−/−^), elicit anti-tumor effects via IFNγ and CXCL10 induction. Due to this bacterial strain lacking endotoxin, the EVs it produces also lack this immunostimulatory cell wall component making this anti-tumor treatment free from adverse side effects in a mouse model.^60^ Furthermore, Gram-positive bacteria show promise with anti-tumor therapy. *S. aureus* EV treatment shows significant long-term anti-tumor effects. In fact, five weeks after treatment with *S. aureus* EVs in mice, the tumor suppression was sustained, and there was no evidence of tumor regeneration events indicating a permanence to this type of treatment.^60^ Additionally, *Lactobacillus acidophilus* and *Lactobacillus rhamnosus*, both considered “good bacteria”, possess anti-tumor activity and cytotoxic effects on cancerous cell lines lending potential to the prospect of using EVs isolated from bacteria already colonized in the human microbiome for clinical applications.^60^,^61^

There is also evidence for the use of EV immunotherapy in combination with other proven cancer therapies to treat aggressive tumors. For example, a low dose of *Salmonella typhimurium* EVs leads to tumor suppression via elevated levels of anti-tumor cytokines while simultaneously triggering leakage of red blood cells (extravasation) into the tumor resulting in a darkened color. This decrease in size and change in pigment renders the tumor more susceptible to phototherapy and irradiation, making this combination treatment extremely successful in tumor eradication.^62^

### 10.2. Pathogen EVs as inflammatory therapeutics

Combination therapies involving natural membranes from bacterial EVs to coat synthetic nanoparticles have also been used in research exploring antibacterial vaccines. A blend of the outer membrane of EVs from *E. coli* (BM) coated onto small gold nanoparticles (AuNPs) generates a BM-AuNP that can be infected subcutaneously to induce the activity and development of innate immune cells in the lymph nodes. Furthermore, these particles induce IFNγ and IL-17, indicating a strong Th1 and Th17 response. This BM-AuNP injection generates antibody responses that are more robust and long-lasting than an EV vaccination alone, suggesting that this combination therapy is effective in the vaccination against the source bacteria.^63^

EVs isolated from parasites and helminths possess clinical potential, especially for diseases that involve hyperinflammation (*e.g*., colitis). Using a dextran sulfate sodium (DSS)-induced colitis model in mice, EVs extracted from parasites reduce the pathologic symptoms of colitis by dampening the pro-inflammatory response and rewiring the innate immune cell activity and signaling cascades. Specifically, EVs from *Fasciola hepatica* alter the immune response to protect against hyperinflammation by reducing pro-inflammatory cytokines and disabling the MAPK and NFκB innate immune signaling pathways.^64^ Similarly, EVs from *N. brasiliensis* protect against colitis-induced inflammation via suppression of key colitis-associated cytokines: IL-6, IL-1β, and IL-17A and simultaneous induction of anti-inflammatory IL-10.^42^ Some parasite-derived EVs protect from colitis by altering the polarization and subsequent activity of innate immune cells like macrophages. *T. spiralis* EVs trigger the increased recruitment of M2 macrophages into the lymph nodes and colons of DSS-treated mice. These colons also showed increased expression of IL-4, IL-10, TGF-β, and IL-13 with coinciding lack of canonical pro-inflammatory cytokines.^43^ The immunomodulatory ability of these various parasitic EVs in a colitis model provides hope for the use of these EVs in the clinic to rewire the host immune system and restore the balance of cytokines and signaling in the gut.

Research into the clinical relevance of EVs isolated from fungal pathogens is still in its infancy. Many studies demonstrating the immunomodulatory potential of fungal EVs focus on vaccination strategies simply using pre-treatment with source fungal EVs. Using the wax moth infection model, *G. mellonella*, pre-treatment with EVs isolated from relevant fungal pathogens like *C. albicans*, *C. neoformans*, and *A. flavus* led to decreased fungal burden in the organism and increased larval survival.^46^,^50^,^51^ Some studies have shown this pre-treatment effective in a murine model of candidiasis, revealing decreased fungal burden in the spleen, liver, and kidney of mice given *C. albicans* EVs prior to infection and increased survival compared to non-vaccinated mice.^52^ Due to the recent advances in understanding the mechanism by which some fungal EVs activate the host innate immune response, there is potential for harboring these immunomodulatory effects for the treatment of other diseases like cancer and colitis, but more work is needed to elucidate the specific ways in which fungal EVs interact with their host.^57^

## 11. Conclusion

Upon pathogenic infection of a host organism, there is a tug-of-war like interaction where dominance over health status of the host toggles between host or pathogen immunomodulatory forces. It has now been elucidated that EVs often serve as the mediator of these interactions from the pathogen side via the delivery of messages that can either elicit a pro-, anti-, or complex inflammatory response. Bacteria, parasites, and fungi all deploy EVs not only as means of inter-species communication but also as unintentional signals and vehicles of infection. Certain bacterial and parasitic species release EVs that shift the health of the host towards a disease state to one more compatible with microbial survival within the host through the induction of anti-inflammatory immune responses. The specific anti-inflammatory response is dependent upon the infectious agent but may include modulation of cytokine release, microbial clearance capacity, host cell survival, and classic inflammatory signaling pathways. Conversely, immune cells can combat EVs released by other pathogenic species and elicit robust pro-inflammatory immune responses through activation of signaling pathways like the MAPK and NFκB pathways and proinflammatory cytokine release. The findings presented in this review revealing the numerous, and sometimes opposing, roles of EVs derived from pathogens demonstrate the vast complexity of the interactions between microorganisms and cells that constitute a host’s first line of immune defense. While specific immunomodulatory cargo in EVs of few pathogenic species have been identified as essential for pathway activation in host cells, more research will be important for further characterization of patterns of host immune cell inflammatory pathways in response to pathogen-derived EVs and to further interpret the puzzle of the dual roles of EVs.

## Figures and Tables

**Table 1:** Characterization of inflammatory responses elicited in host innate immune cells in response to bacteria-derived EVs.

Bacterial Species	Pro-inflammatory responses		Anti-inflammatory responses	
	Cell Type	Response	Cell Type	Response
*Lactiplantibacillus plantarum*	Murine macrophages	IL-1β, IL-6, IL-12 & IFNγ production^15^ NFκB signaling via TLR2^15^	Murine macrophages	IL-10 production^15^
*Staphylococcus aureus*	Murine macrophages Murine macrophages Murine splenocytes	IFNβ mRNA production^16^ MIP-2, IL-6, & TNFα production^17^ MIP-2, IL-6, & TNFα production^17^		
*Francisella tularensis*	Murine macrophages	Caspase activity reduction^2^ TNFα, IL-6, IL-12p70, CXCL1, MCP-1, & IL-1α production^2^	Murine macrophages	IL-10 upregulation^2^ Membrane disruption^2^ Increased caspase activity^2^
*Listeria monocytogenes*	Human intestinal epithelial cells	TNFα, NOD-like receptor, IL-17, NFκB, MAPK, relaxin, & PI3K-Akt signaling^19^		
*Fusobacterium nucleatum*	Human intestinal epithelial cells	NFκB signaling^20^		
*Streptococcus pneumoniae*	Murine macrophages	NFκB signaling^21^	*In vivo* murine model	Natural killer, helper T-cell, and
	*In vivo* murine model	Macrophage population expansion, especially in the spleen^21^		cytotoxic T cell reduction^21^
*Helicobacter pylori*			Murine macrophages	IL-4 & IL-10 induction^26^ M2 macrophage polarization^26^ M1 macrophage polarization reduction^26^
*Pseudomonas aeruginosa*	Mouse primary adipose tissue derived mesenchymal stem cells	cGAS-STING pathway^24^ IL-7 production^24^	Human lung macrophages	MHC gene suppression^25^ IL-10 induction^25^
*Neisseria gonorrhoeae*			Murine macrophages	Apoptosis^27^ Mitochondrial & plasma membrane damage^27^
*Gardnerella vaginalis*	Vaginal epithelial cells and cervical epithelial cells	IL-6, IL-7, IL-8, IL-13, G-CSF, GM-CSF, MIP-1α induction via NFκB signaling^22^		
*Mobiluncus mulieris*	Human vaginal epithelial cells and cervical epithelial cells	IL-6, IL-7, IL-8, IL-13, G-CSF, GM-CSF, MIP-1α, MIP-1β, & TNFα induction via NFκB signaling^22^		
*Bifidobacterium longum*			Murine splenocytes Murine dendritic cells	IL-10 induction^28^ IL-10 induction^28^
Avian pathogenic *Escherichia coli*	Human macrophages	IL-1β, IL-18, & TNFα production^23^ NFκB signaling via TLR4^23^ NLRP3 inflammasome activation^23^	Human macrophages	Apoptosis activation^23^
	Murine neutrophils	NET formation^23^ SAPK/JNK signaling^23^		
*Vibrio cholerae*			Human intestinal epithelial cells	Anti-inflammatory microRNA induction^3^ CCL20 and IL-18 mRNA reduction^3^

**Table 2:** Characterization of inflammatory responses elicited in host innate immune cells in response to protozoan parasite-derived EVs.

Protozoan Parasite Species	Pro-inflammatory responses		Anti-inflammatory responses	
	Cell Type	Response	Cell Type	Response
*Trypanosoma cruzi*	Murine macrophages	TNFα, IL-6, & NO production^29^ MAPK signaling^29^	Murine Macrophages	IL-12p40, IL-6, and TNFα downregulation via TLR2-AKT pathway^29^
	*In vivo* murine splenocytes	TNFα, IFNγ, & IL-6 production NO increase^29^	*In vivo* murine splenocytes	IL-10 production^29^
*Trypanosoma evansi*			Murine macrophages	IL-12p40, IL-6, and TNFα reduction^36^
*Giardia duodenalis*	Murine peritoneal macrophages	IL-1β, IL-6, IL-12, IL-17, IFN-γ, TNF, IL-18, CCL20, & CXCL2 via TLR2^30^,^35^ NLRP3 inflammasome signaling activation^30^ MAPK and NFκB signaling^35^	Murine peritoneal macrophages	IL-10 production^30^ Akt signaling^35^
*Toxoplasma gondii*	Murine macrophages	TNFα & iNOS production^31^	Murine macrophages	IL-10 production^31^
*Trypanosoma brucei*	Murine macrophages	NO & urea production^32^ MHC expression upregulation^32^		
*Eimeria falciformis*	Murine Intestinal Epithelial Cells	IL-1β, IL-6, IL-17, & IL-18 production^33^	Murine Intestinal Cells	Pyroptosis^33^
*Entamoeba histolytica*	Human neutrophils	ROS production^13^	Human neutrophils	NET & respiratory burst reduction^13^
*Leishmania infantum*	Human macrophages	Macrophage motility activation^37^	Human macrophages	IL-10production & IL-8 reduction^37^
*Naegleria fowleri*	Murine microglial cells	TNFα, IL-1α, IL-1β, IL-6, IL-17, IFNγ, MIP-1α & MIP-2 production via TLR2/TLR-4^34^ MAPK signaling^34^ NFκB signaling^34^ JAK-STAT signaling^34^		

**Table 3:** Characterization of inflammatory responses elicited in host innate immune cells in response to helminth-derived EVs.

Helminth Species	Pro-inflammatory responses		Anti-inflammatory responses	
	Cell Type	Response	Cell Type	Response
*Clonorchis sinensis*	*In vivo* Murine hepatic macrophages	TNFα & IL-6 production^38^ M1 macrophage polarization^38^ NFκB signaling^38^		
*Brugia malayi*	Murine macrophages	TNFα, IL-6, MCP-1, MIP-2, G-CSF, LIX, & RANTES production^39^ M1 macrophage polarization^39^	Human dendritic cells	mTOR signaling downregulation^8^
*Schistosoma mansoni*	Human umbilical vein endothelial cells	TNFα, IL-6, CXCL2, CXCL11, TNFR, IL-15R, & IL-21R gene upregulation^40^		
*Heligmosomoides polygyrus*			Murine macrophages	Macrophage suppression^41^ IL-33 pathway inhibition^41^
*Nippostrongylus brasiliensis*			*In vivo murine* colon cells	IL-6, IL-1β, IFNγ, IL-17a suppression^42^ IL-10 production^42^
*Trichinella spiralis*			*In vivo* murine colon cells	TNFα, IFNγ, IL-17a, IL-1β downregulation^43^ M1 macrophage polarization inhibition^43^ M2 macrophage polarization activation^43^ MAPK signaling reduction^43^

**Table 4:** Characterization of inflammatory responses elicited in host innate immune cells in response to fungal-derived EVs.

Fungal Species	Pro-inflammatory responses		Anti-inflammatory responses	
	Cell Type	Response	Cell Type	Response
*Candida albicans*	Murine macrophages	cGAS-STING pathway & IFNβ production^57^ IL-12, TGF-β, TNFα, & NO production MHCs upregulation^50^ CCL2 production^53^ Increase phagocytic and fungicidal ability against whole organism^53^		
	*In vivo* murine models	Reduce keratitis and fungal load of whole organism^53^		
	Human macrophages	IL-8 & TNFα production & IL-10 reduction^44^		
	Human neutrophils	Pro-inflammatory cytokine & NO production^53^ Increase phagocytic and fungicidal ability against whole organism^53^		
*Candida auris*	Murine dendritic cells	IL-6 production^45^ Dendritic cell activation^45^		
*Candida glabrata*	Human monocytes	TNFα & IL-8 production & IL-10 reduction^47^		
*Candida tropicalis*	Human monocytes	TNFα & IL-8 production & IL-10 reduction^47^		
*Candida parapsilosis*	Human monocytes	TNFα & IL-8 production & IL-10 reduction^47^		
*Aspergillus flavus*	Murine macrophages	IL-6, IL-1β, TNFα, & NO production^46^ M1 macrophage polarization^46^ Increased macrophage phagocytic capacity^46^		
*Aspergillus fumigatus*	Murine macrophages	Increased fungal clearance capacity^54^ TNFα and CCL2 production^54^ TNFα production & NO synthase & adhesion molecule gene expression^58^		
	Murine neutrophils *in vivo Galleria Mellonella* model	Increased killing of whole microorganism^54^ Increased host survival to whole organism challenge^58^		
*Cryptococcus neoformans*	Murine macrophages	Increased phagocytic and microbicidal activity^55^ Macrophage EV release that activate p53 and mTOR signaling in distant macrophages^56^	IL-10 & TGF-β production^55^	
	Human macrophages	Macrophage EV release that activate p53 and mTOR signaling in distant macrophages^56^		
*Paracoccidioides brasiliensis*	Murine macrophages *In vivo* murine model	TNFα, IL-6, MCP-1, & NO production^48^ Increased lung CFU of whole organism Circulating IL-6, IFNγ, MCP-1, IL-6 increase^48^	*In vivo* murine model	NO & IL-6 reduction in lung^48^
*Talaromyces marneffei*	Murine macrophages	ROS, NO, IL-1β, IL-6, TNFα production^59^	Murine macrophages	IL-10 production^59^
	Human macrophages	IL-1β & IL-6 production^49^ Macrophage M1 polarization^49^ Increased phagocytosis ability of whole organism^49^	Human macrophages	IL-10 production^49^
